# Circular RNAs: Promising Treatment Targets and Biomarkers of Ischemic Stroke

**DOI:** 10.3390/ijms25010178

**Published:** 2023-12-22

**Authors:** Guangchen Xu, Ge Liu, Ziyu Wang, Yunman Li, Weirong Fang

**Affiliations:** Department of Physiology, School of Basic Medicine and Clinical Pharmacy, China Pharmaceutical University, Nanjing 210009, China; 3221091977@stu.cpu.edu.cn (G.X.); 3121090285@stu.cpu.edu.cn (G.L.); ziyuwang@stu.cpu.edu.cn (Z.W.)

**Keywords:** CircRNA, ischemic stroke, miRNA sponge, treatment target, biomarker

## Abstract

Ischemic stroke is one of the most significant causes of morbidity and mortality worldwide. However, there is a dearth of effective drugs and treatment methods for ischemic stroke. Significant numbers of circular RNAs (circRNAs) exhibit abnormal expression following ischemic stroke and are considered potential therapeutic targets. CircRNAs have emerged as promising biomarkers due to their stable expression in peripheral blood and their potential significance in ischemic stroke diagnosis and prognosis. This review provides a summary of 31 circRNAs involved in the pathophysiological processes of apoptosis, autophagy, inflammation, oxidative stress, and angiogenesis following ischemic stroke. Furthermore, we discuss the mechanisms of action of said circRNAs and their potential clinical applications. Ultimately, circRNAs exhibit promise as both therapeutic targets and biomarkers for ischemic stroke.

## 1. Introduction

Circular RNAs (circRNAs) are a class of non-coding RNA molecules that lack a 5′ cap and 3′ poly (A) tail and form a circular structure through covalent bonds. Initially discovered in plant viruses, they were thought to be a byproduct of weakly functioning abnormal splicing [1], and in 1979, were detected in human Hela cells [2], followed by prokaryotes, single-celled eukaryotes, and mammals. With the development of high-throughput sequencing technology, research on circRNAs is rapidly increasing. CircRNAs are produced by the back-splicing of exons, introns, or both [3]. After transcription in the nucleus, circRNAs are transferred to the cytoplasm, where they mainly exert physiological functions, but can also be secreted into body fluids via exosomes, subsequently entering other tissue cells to exert their effects [4]. Existing studies have shown that circRNAs can participate in the regulation of neuronal function [5], immunity [6], and the blood–brain barrier (BBB) [7], functioning in the acute stage after ischemic stroke along with post-stroke brain injury recovery.

The prevalence of stroke in China was 2.6%, with 505.2 new patients per 100,000 population in 2020 [8]. Stroke is an acute cerebrovascular disease caused by the sudden rupture or blockage of blood vessels in the brain, resulting in brain tissue damage. Stroke is divided into ischemic stroke and hemorrhagic stroke, with ischemic stroke accounting for 86.8% of the total number of strokes in 2020 [8]. Ischemic stroke is mainly caused by the blockage of blood vessels in the brain, with small vessel disease, cardioembolism, and large artery atherosclerosis being the main causes of cerebral vascular occlusion [9]. Reversible neuronal dysfunction occurs first when cerebral vascular occlusion leads to insufficient blood supply to brain tissue. As the ischemic time increases, irreversible neuronal functional damage occurs. Oxidative stress, inflammation, apoptosis, necrosis, and other processes exacerbate the damage to neurological function after ischemic stroke. Methods of mitigating the damage to neurological function caused by these processes are of critical importance. This study reviews the involvement of circRNAs in the regulation of various pathological changes and performance as biomarkers for ischemic stroke.

## 2. Biological Functions of circRNAs

**Figure 1:**
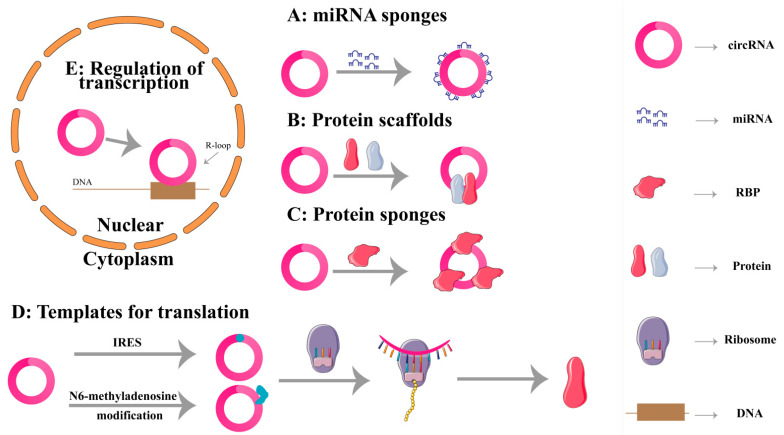
Biological functions of circular RNAs (circRNAs). (**A**) CircRNAs act as miRNA sponges to prevent miRNA from binding and suppressing their target mRNAs. (**B**) CircRNAs act as protein scaffolds to form a circRNA-protein complex and influence protein interactions. (**C**) CircRNAs act as RNA-binding protein (RBP) sponges to prevent RBPs from functioning. (**D**) CircRNAs with internal ribosomal entry sites (IRES) or that have undergone N6-methyladenosine modification can translate proteins. (**E**) CircRNAs regulate transcription by forming an R-loop.

The predominant function of circRNAs identified so far is their role as a sponge for microRNA (miRNA) [10], though they also serve a role in transcriptional regulation and can directly bind to RNA-binding proteins (RBPs) to regulate mRNA stability [11]. Moreover, circRNAs can serve as scaffolds to influence protein–protein interactions at the protein level [12]. Recent studies have also demonstrated that circRNAs can undergo cap-independent translation to produce proteins or peptides [13]. We summarize the five biological functions of circRNAs in the following (Figure 1).

### 2.1. miRNA Sponges

The regulatory mechanism of circRNAs as miRNA sponges was first identified by Thomas B. Hansen and colleagues in 2013, thus laying a theoretical foundation for further research on the topic [14]. CircRNAs contain one or more binding sites for miRNA, which competitively bind to miRNA and thereby affect the expression of downstream targets, participating in different physiological and pathological processes of many diseases, such as cancer, stroke, and hypertension [10]. For instance, the combination of circRNA_0088036 and miR-40-3p can promote the expression of the downstream target forkhead box Q1, thereby promoting the proliferation, migration, invasion tumorigenesis, and metastasis of bladder cancer cells [15]. CircBCBM1, is upregulated in the breast cancer brain metastasis cells and clinical tissue and plasma samples, acting as the sponge of miR-125a to breast cancer brain metastasis via the miR-125a/BRD4 axis [16]. The miRNA sponge function of circRNAs may be the main mechanism for their physiological functions.

### 2.2. Protein Scaffolds

CircRNAs can act as a scaffold to connect two proteins and enhance or dissociate their interaction [12]. CircRNAs-CREIT binds to protein kinase (PKR) and E3 ubiquitin protein ligase 1 (HACE1), enhancing PKR degradation through the HACE1-mediated ubiquitin-proteasome pathway. Consequently, it inhibits the assembly of stress granules and activates the receptor for the activated C kinase 1 (RACK1)/MAP three kinase 1 (MTK1) apoptosis signaling pathway [17], thereby inhibiting triple-negative breast cancer (TNBC) tumor growth and reducing the resistance of TNBC cells to doxorubicin. CircCCNB1 binds to Cyclin B1 (Ccnb1) and Cyclin-dependent kinase 1 (Cdk1) to dissociate the Ccnb1/Cdk1 complex, causing Ccnb1 to lose its ability to promote cell migration, invasion, proliferation, and survival [18]. At present, there are few studies on the involvement of circRNAs in the pathophysiological process of ischemic stroke through the function of protein scaffolds.

### 2.3. Protein Sponges

CircRNAs can competitively bind with and sequester RBPs, impeding RBPs from performing their biological functions, such as affecting the stability of mRNA [11]. For instance, circPPFIA1s has a binding site on Hu antigen R (HuR). Upon binding with circPPFIA1s, HuR becomes unable to bind with the mRNA of the Member RAS oncogene family 36 (RAB36). Consequently, the stability of RAB36 mRNA decreases, leading to a reduction in RAB36 protein expression, which inhibits the metastasis of colorectal cancer [19]. In another case, circTHBS1 can strengthen the HuR-mediated mRNA stability of INHBA, which subsequently activated the TGF-β pathway [20].

### 2.4. Translation

Although circRNAs lack conventional ribosome binding sites for regular translation, certain circRNAs with internal ribosomal entry sites (IRES) can be translated through a cap-independent mechanism [21]. IRESs are sequences that recruit initiation factors or ribosomes to mRNA for cap-independent translation. In Drosophila, for example, circMbl has been found to be capable of being translated into a protein by IRES [22]. Similarly, in mice, circ-ZNF609 has been shown to undergo translation and participate in the myogenesis process [23]. Additionally, circRNAs can also be modified through N6-methyladenosine to translate functional protein [24]. N6-methyladenosine is rich in circRNAs and a single N6-methyladenosine site is sufficient to drive translation initiation [24]. However, the translation mechanism of N6-methyladenosine is not clear. An instance of this is observed in circMAP3K4, where N6-methyladenosine modification led to the encoding of circMAP3K4-455aa, a protein that plays a role in preventing apoptosis in hepatocellular carcinoma [25]. These examples emphasize the diverse and intricate ways in which circRNAs can participate in cellular processes by being translated into functional proteins, thereby expanding our understanding of their biological roles beyond their traditional function as miRNA sponges. However, due to the low efficiency of cap-independent translation, the abundance of proteins translated by circRNAs is low, and whether the proteins are useful remains to be investigated.

### 2.5. Regulating Transcription

CircRNAs, located in the cell nucleus, play a role in regulating the transcription process [26]. For example, circSMARCA5 in breast cancer has binding sites on its parental gene and, upon binding, forms an R-loop that pauses transcription at exon 15 of SMARCA5, a member of the SWI/SNF complex with ATP-dependent chromatin remodeling activity, resulting in decreased expression of SMARCA5 and the production of truncated non-functional protein [27]. Similarly, circZNF827 can form a nuclear transcriptional repression complex by binding to hnRNP-K/L to inhibit transcription of nerve growth factor receptor (NGFR) and negatively regulate neuronal differentiation [28]. In addition, circRNAs can compete with linear splicing to regulate transcription, as seen with circMBL [29]. The diverse mechanisms by which circRNAs can modulate transcriptional processes in the cell nucleus add another layer of complexity to their regulatory roles in gene expression.

## 3. CircRNAs and Stroke

CircRNAs are tissue-specific and expressed within the central nervous system. Existing studies have shown that circRNAs are closely related to neurological impairment and may become a new target and direction for ischemic stroke. The role of circRNAs in ischemic stroke is presented in the following aspects: apoptosis, autophagy, inflammation, oxidative stress, and angiogenesis (Table 1).

### 3.1. Apoptosis

Apoptosis is a genetically controlled, autonomous, and orderly way of cell death formed in the process of biological evolution. Upon apoptosis, cell volume shrinks, cytoplasmic density increases, the nuclear cytoplasm condenses, the nuclear membrane and nucleolus are fragmented, and finally apoptotic bodies are formed. Apoptosis can be triggered by extrinsic pathways and intrinsic pathways [65]. Intrinsic pathways are induced by the expression of apoptosis—inducing factors and cytochrome C in the cytosol when mitochondria are destroyed by toxic agents or DNA damage. Alternatively, the activation of death receptors (Fas/FasL) on the cell membrane can trigger extrinsic pathways [66]. In the human body, B-cell lymphoma-2 (Bcl-2) is a gene that inhibits apoptosis, whereas Bcl-2 associated X protein (Bax) is the most important apoptotic gene. The inhibitory effect of Bcl-2 on apoptosis can be counteracted by the overexpression of Bax [67]. Caspase3 can be activated to promote DNA cleavage that leads to apoptosis [68]. When stroke occurs, the expression of Bax and cleaved caspase3 (C-caspase3) increases, while the expression of Bcl-2 decreases and promotes cell apoptosis. The “abnormal” apoptosis of cells is induced under circumstances of massive releases of reactive oxygen species and DNA and mitochondrial damage. Apoptosis in the penumbra region seems to be reversible [69], discovering new targets that can inhibit apoptosis is one of the most important tasks to mitigate stroke damage.

#### 3.1.1. CircFOXP1

CircFOXP1 is obtained by reversing the splicing of exons 3, 4, 5, and 6 of the FOXP1 precursor mRNA. In patients with acute ischemic stroke, decreased expression of circFOXP1 in peripheral blood is observed. Overexpression of circFOXP1 in transient middle cerebral artery occlusion (tMCAO) mice and glucose deprivation/reoxygenation (OGD/R)-treated human glioblastoma cells (A172) could inhibit apoptosis signal transduction and reduce cerebral ischemic injury. CircFOXP1 is able to bind to signal transducer and activator of transcription 3 (STAT3), which is a member of the anti-apoptotic signaling cascade [70], thereby reducing ubiquitination and degradation of the STAT3 protein and ultimately inhibiting the transduction of the apoptotic signaling pathway [30].

#### 3.1.2. CircCDC14A

CircCDC14A is upregulated after ischemic stroke and can act as a sponge of miR-23a-3p to regulate the expression of chemokine stromal-derived factor-1 (CDCL12). CDCL12, a member of the CXC chemokine subfamily, induces the inactivation of caspase3 by reducing the ratio of Bax to Bcl-2. When circCDC14A is knocked out in middle cerebral artery occlusion/reperfusion (MCAO/R) mice and OGD/R-treated hippocampal neuronal cell line (HT22 cells), apoptosis is inhibited through activating the circCDC14A/miR-23a-3p/CDCL12 axis [31]. In addition, recent studies had shown that circCDC14A, when silenced in peripheral blood, could alleviate the inflammatory damage through inhibiting astrocytes activation caused by ischemic stroke [71].

#### 3.1.3. CircOGDH

CircOGDH, derived from exons 3 and 4 of the OGDH mRNA, was significantly upregulated in the peripheral blood and brain injury penumbra of patients with acute cerebral ischemia [32]. In middle cerebral artery occlusion (MCAO) mice, circOGDH was enriched primarily in neurons in the penumbra region. In vitro, circOGDH was found to be predominantly present in the cytoplasm. As the miR-5112 molecular sponge, circOGDH had a positive effect on COL4A4 (Gallus collagen, type IV, alpha IV), which was the downstream target of miR-5112. Knockout of circOGDH alleviated neuronal injury in the penumbra area and inhibited apoptosis in primary cortical neurons in MCAO mice. Mechanistically, knockout of circOGDH enlarges the BCL-2/BAX ratio and inhibits the activity of C-caspase3 through the miR-5112/COL4A4 axis [32].

#### 3.1.4. CircPRDX3

CircPRDX3 acted as the sponge of miR-641 and negatively regulated its expression while increasing the expression of Natriuretic Peptide Receptor 3 (NPR3). Expression of circPRDX3 was downregulated in MCAO/R mice and OGD/R-treated neuroblastoma adipose tissue of mice Neuro2a (N2a) cells. NPR3, a constituent of the natriuretic peptide receptor, was the downstream target of miR-641. NPR3 was found to inhibit members of the mitogen-activated protein kinases (MAPK) pathway which is the apoptosis related pathway [33]. Overexpression of circPRDX3 can inactivate the MAPK pathway to inhibit apoptosis after stroke through the circPRDX3/miR-641/NPR3 axis [33].

#### 3.1.5. Circ_0000566

Circ_0000566 and Activin A Receptor type IIB (ACVR2B) was upregulated in OGD/R-treated human brain microvascular endothelial cells (HBMECs). A previous study showed ACVR2B contributes to ischemic neuronal injury via the Smad2/c-jun axis [72]. Recent studies demonstrate that silencing circ_0000566 lead to a decrease in the expression levels of ACVR2B, Bax, and C-caspase 3 in OGD/R-treated HBMECs, and the expression level of Bcl-2 increased, indicating that circ_0000566 could promote apoptosis of HBMECs and aggravate cell damage [34]. Mechanistically, circ_0000566 acted as a sponge to positively regulate the downstream target of miR-18a-5p, ACVR2B, thereby modulating the expression of apoptotic-related molecules. In summary, silencing circ_0000566 can alleviate OGD/R-induced damage to HBMECs and inhibit apoptosis of HBMECs through the miR-18a-5p/ACVR2B axis [34].

#### 3.1.6. Circ_0000647

Circ_0000647, derived from the SEC11A gene, has a fragment length of 380nt. After OGD/R treatment of the human neuroblastoma cell line (SK-N-SH cells), the expression of circ_0000647 was found to be upregulated. Meanwhile, knocking down circ_0000647 inhibited the apoptosis of SK-N-SH cells and promoted cell proliferation. Mechanistically, circ_0000647 acted as a molecular sponge to bind to miR-126–5p and reduce its expression, which led to an increase in the expression of TNF receptor associated factor 3 (TRAF3), the target of miR-126–5p. This cascade promoted apoptosis and inhibited the proliferation of SK-N-SH cells. Circ_0000647 promotes apoptosis in SK-N-SH cells through the miR-126–5p/TRAF3 axis and may be a new target for stroke treatment [35].

#### 3.1.7. CircFUNDC1

The expression of circFUNDC1 (circ_0007290), which is transcribed from FUN14 Domain Containing 1 (FUNDC1), was increased in the plasma of acute ischemic stroke patients. CircFUNDC1 acted as a sponge of miR-496 and upregulated the expression of the downstream target programmed cell death protein 4 (PCDC4). PCDC4 is the tumor suppressor and protein translation inhibitor and is expressed in aggravated cell damage of OGD/R-treated cells [73]. In recent studies, silencing circFUNDC1 expression in OGD-treated human cortical neurons (HCN-2 cells), inhibited apoptosis through the circFUNDC1/miR-496/PCDC4 axis [36]. The expression levels of C-caspase3 and cleaved caspase9 (C-caspase9) were reduced, which also indicated that silencing circFUNDC1 could suppress the apoptosis of HCN-2 cells.

#### 3.1.8. CircTLK1

CircTLK1 is derived from the second chromosome and is approximately 256 nucleotides in length. As a molecular sponge of miR-26a-5p, circTLK1 enhanced the expression level of insulin-like growth factor type 1 receptor (IGF-1 R) and glucose transporter type 1 (GLUT1) via the miR-26a-5p/tensin homolog (PTEN)/IGF-1 R/GLUT1 axis. Recent studies demonstrate that knockdown of circTLK1 in mice and N2a cells decreased the expression of C-caspase3 and Bax by IGF-1 R and GLUT1, reducing apoptosis [37]. Therefore, knocking out the circTLK1 gene can reduce the apoptosis of post-stroke brain tissue cells, alleviate brain damage, and improve cell viability through the miR-26a-5p/PTEN/IGF-1R/GLUT1 axis [37].

#### 3.1.9. CircUSP36

CircUSP36, transcribed from the gene USP36, was significantly reduced in the peripheral blood of IS patients, and functioned as a molecular sponge of miR-139–3p which could negatively regulate SMAD family member 3 (SMAD3) expression. SMAD3 is a member of the SMAD protein family and has been reported to promote Bcl-2 expression and inhibit neuronal apoptosis in MCAO rats [74]. In vitro, circUSP36 has been shown to inhibit astrocyte apoptosis in OGD/R models. In vivo, overexpression of circUSP36 promoted SMAD3 and Bcl-2 expression and inhibited astrocyte apoptosis in MCAO/R mice. In short, circUSP36 inhibits astrocyte apoptosis and reduces neuronal damage after stroke through the miR-139–3p/SMAD3/Bcl-2 axis [38].

#### 3.1.10. CircHIPK3

CircHIPK3 is encoded by the homeodomain-interacting protein kinase 3 (HIPK3) gene, whose expression was suppressed in tMCAO mice. It functioned as the molecular sponge of miR-148b-3p, demonstrating positive effect on cyclin-dependent kinase 5 regulatory subunit 1 (CDK5R1), which was a downstream target of miR-148b-3p. CDK5R1 can bind to cyclin-dependent kinase 5 (CDK5), which inhibits silent information regulator 1 (SIRT1) expression. SIRT1, in turn, increases the expression of Bcl-2 and suppressed the expression of Bax, thereby inhibiting cell apoptosis and mitochondrial dysfunction [75]. Overexpression of circHIPK3 in the OGD/R model of brain-derived Endothelial cells.3 (bEnd.3) and tMCAO mice inhibited cell apoptosis and mitochondrial dysfunction by promoting SIRT1 expression through the circHIPK3/miR-148b-3p/CDK5R1/SIRT1 signaling pathway [39].

#### 3.1.11. Circ_0010729

Circ_0010729 was the molecular sponge of miR-665, which negatively regulates the expression of growth family member 5 (ING5). ING5 is involved in many cellular activities such as DNA replication and apoptosis. It has been shown to promote apoptosis in newborns after stroke [76]. Circ_0010729 expression was upregulated in the OGD/R model of HBMECs. Inhibiting the expression of circ_0010729 could decrease the expression of ING5, Bax, and caspase3 while increasing the expression of Bcl-2, thus inhibiting the apoptosis of HBMECs. In summary, the knockdown of circ_0010729 attenuates apoptosis in OGD/R treated HBMECs through the miR-665/ING5 axis [40].

#### 3.1.12. CircPUM1

Expression of circPUM1, which is derived from the exonic back-splicing of the PUM1 gene, was decreased after cerebral ischemia/reperfusion injury. As the sponge of miR-340–5p, circPUM1 positively regulated its downstream target DEAD box helicase 5 (DDX5), which belongs to the DEAD-box RNA helicase family and inhibits TNF-α-mediated apoptosis [77]. Overexpression of circPUM1 reduced apoptosis and inflammation, thereby reducing neuronal cell damage after stroke. This mechanism was achieved through the miR-340–5p/DDX5 axis, where circPUM1 overexpression promotes DDX5 expression and inhibits TNF-α-mediated apoptosis [41].

#### 3.1.13. CircHECTD1

CircHECTD1 is the sponge of miR-133b which was translated from the HECTD1 gene and is located on exons 23 and 24 of the HECTD1 gene. TRAF3, a downstream intracellular signaling molecule of miR-133b, belongs to the TNFR family [78]. Knocking down circHECTD1 upregulated the expression of miR-133b and downregulated the expression of TRAF3, leading to the inhibition of apoptosis and alleviation of cell damage by reducing C-caspase3 and NF-κB p65 in MCAO mice and OGD-treated HT22 cells [43]. In addition, circHECTD1 serves as a sponge for miR-27a-3p which negatively regulates follistatin-like 1 (FSTL1). FSTL1 is an extracellular glycoprotein found in nature that plays a role in cardiovascular disease and inflammation [79]. In MCAO mice and OGD-treated HT22 cells, circHECTD1 and FSTL1 were highly expressed and miR-27a-3p was downregulated. Knockdown of circHECTD1 inhibited cell apoptosis and reduced cell damage after stroke through the miR-133b/TRAF3 axis and miR-27a-3p/FSTL1 axis [42,43]. Other studies have shown that knocking out circHECTD1 inhibits astrocyte autophagy through MIR142/TIPARP axis and alleviates cerebral ischemic injury [80].

#### 3.1.14. CircPHC3

CircPHC3 was found to be abundant in neonatal umbilical cord plasma and encoded by the PHC3 gene. By acting as the sponge for miR-455–5p, circPHC3 positively regulated TRAF3, a downstream target of miR-455–5p. In OGD-treated HBMECs, circPHC3 was overexpressed. Silencing circPHC in OGD-treated HBMECs reduced mi-455–5p expression and over-expressed TRAF3. Flow cytometry assay illustrated that cell apoptosis of HBMECs was suppressed significantly. These functions were mediated through the miR-455–5p/TRAF3 axis [44].

#### 3.1.15. CircTTC3

CircTTC3 was upregulated in OGD/R-treated primary astrocytes and MCAO/R mice. As a molecular sponge of miR-372–3p, circTTC3 directly participated in the regulation of Toll-like receptor 4 (TLR4) which was the downstream target of miR-455–5p. Knocking out circTTC3 could reduce the cerebral infarct size, neurological score, and brain water content after ischemic stroke, inhibit the apoptosis of astrocytes, and promote the proliferation and upregulation of expression of nestin and β-tubulin III in neural stem cells. The expression of miR-372–3p and Bcl2 was upregulated after knockdown of circTTC3. In contrast, the expression of TLR4, Bax, and C-caspase 3 was downregulated. In summary, silencing circTTC3 can inhibit the apoptosis of astrocytes and reduce cell damage through the miR-372–3p/TLR4 axis [45].

#### 3.1.16. CircCCDC9

CircCCDC9 is derived from exons 6 and 7 of the CCDC9 gene and was downregulated in the brain tissue of tMCAO mice. Activation of the Notch pathway aggravates neuronal damage in ischemic stroke by increasing vulnerability to apoptosis [81]. Inhibition of the Notch pathway leads to the downregulation of Notch1 and Notch intraCellular domain (NICD) expression when circCCDC9 was overexpressed. Caspase-3 expression and the ratio of Bax/Bcl2 were reduced. Knockdown of circCCDC9 in tMCAO mice had the opposite effects. Therefore, circCCDC9 attenuates apoptosis after tMCAO through the Notch pathway [46]. However, the interaction between circCCDC9 and Notch1 has not been elucidated. Furthermore, overexpressing circCCDC9 also mitigated blood–brain barrier damage by suppressing the Notch signaling pathway [46].

#### 3.1.17. CircUCK2

The expression of circUCK2 was inhibited in OGD-treated HT-22 cells and brain tissue cells of MCAO mice. circUCK2 was the sponge of miR-125b-5p, which negatively regulated growth differentiation factor 11 (GDF11). GDF11 promotes the expression of Smad3, which inhibits cell apoptosis and reduces brain injury after stroke by activating the TGF-β/Smad3 signaling pathway [82]. When circUCK2 expression was upregulated, the expression of GDF11 and Smad3 significantly increased. CircUCK2 can activate the TGF-β/Smad3 signaling pathway through the miR-125b-5p/GDF11 axis in order to reduce cell apoptosis and improve neuronal injury after stroke [47].

#### 3.1.18. Circ_016719

Circ_016719 was significantly upregulated in tMCAO mice and HT22 cells treated with OGD/R. As a molecular sponge of miR-29c, circ_016719 accelerated the expression of Rac-MAPK kinase 6 (Map2k6), the downstream target of miR-29c. In tMCAO mice and HT22 cells, Map2k6 and p38 was upregulated. Map2k6, also known as MKK6, is a specific activator of p38; the Map2k6/p38 pathway promotes apoptosis [83,84]. When circ_016719 was knocked out, expression of Map2k6 and p38 decreased while expression of miR-29c increased. All in all, knocking down circ_016719 can inhibit apoptosis through the miR-29c/Map2k6 axis after cerebral ischemia/reperfusion injuries [48]. Additionally, significant increases in MAP1LC3B–I and p62 levels and decreased MAP1LC3-II levels were observed, indicating that circ_016719 knockdown had significantly inhibited autophagy.

#### 3.1.19. CircLIFR

Has_circ_0072309 (circLIFR), encoded by the exon of the LIFR gene, exhibited decreased expression in the peripheral blood of patients with ischemic stroke. circLIFR acted as a miR-100 sponge and positively regulated the expression of the downstream target mammalian target of rapamycin (mTOR). The expression of p-mTOR increased significantly and C-caspase3 reduced upon overexpression of circLIFR in MCAO mice and OGD-treated bEnd.3 cells. At this point, apoptosis was inhibited, and cell viability was promoted. Therefore, circLIFR can inhibit apoptosis and reduce cerebral ischemic injury via the miR-100/mTOR axis [49].

#### 3.1.20. Circ_0025984

Circ_0025984 is enriched in the hippocampus of the brain, but its expression was decreased in MCAO rats. circ_0025984 negatively regulated the expression of miR-134–3p through the molecular sponge effect. Meanwhile, miR-134–3p inhibited the expression of its downstream target, ten-eleven translocation methylcytosine dioxygenase 1 (TET1), which can promote the expression of 150-kDa oxygen-regulated protein (ORP150) through demethylation. ORP150 is an endoplasmic reticulum stress marker, which can inhibit the process of endoplasmic reticulum stress to inhibit cell apoptosis [85]. Up-regulation of circ_0025984 expression in OGD-treated A172 cells and MCAO rats could inhibit the apoptosis of astrocytes and reduce the area of cerebral infarction. Consequently, circ_0025984 can inhibit the apoptosis of astrocytes through the miR-134–3p/TET1/ORP150 axis [50].

#### 3.1.21. CDR1as

The expression of circRNAs CDR1as(CDR1as) and miR-7, which are enriched in mouse neurons, were significantly reduced in the brain tissue of MCAO/R mice. CDR1as was a molecular sponge of miR-7 but could not “isolate” miR-7. Instead, it was likely that CDR1as promoted miR-7 stability and acted as a storage chamber for miR-7 [86,87]. MiR-7 directly bound with α-synuclein, which has been shown to aggravate brain injury after stroke and restrain the expression of α-synuclein [88]. After the overexpression of CDR1as in MCAO/R mice, miR-7 expression was upregulated, and α-synuclein expression was downregulated, resulting in reduced apoptosis in neurons [51].

### 3.2. Autophagy

Autophagy is a process of engulfing cytoplasmic proteins or organelles and encapsulating them into vesicles, then fusing them with lysosomes to form autolysosomes and degrade their encapsulated contents, thereby fulfilling the metabolic needs of the cell itself and the renewal of certain organelles. Normal autophagy is of great significance for maintaining cellular homeostasis and eliminating pathogenic microorganisms invading the cytoplasm. However, under ischemic and hypoxic conditions, autophagy is over-activated, which leads to cell death. Excessive autophagy after stroke is one of the important causes of tissue damage, and some circRNAs can influence autophagy after stroke.

#### 3.2.1. Circ-FoxO3

Circ-FoxO3 was abundantly expressed at the ischemic area in MCAO/R mice and is encoded by the second exon of the FoxO3 gene. It is worth mentioning that human circ-FoxO3 has 90.87% identical arrangement order compared to mice. Mammalian target of rapamycin complex 1(mTORC1) can inhibit autophagy, and its activation itself also requires the assistance of E2F transcription factor 1 (E2F1) [89]. In addition, mTOR is one of the components of mTORC1. As the sponge of RBPs, circ-FoxO3 can bind to mTOR and E2F1 to isolate mTOR and E2F1, respectively, thereby inhibiting the expression level of mTORC1. In the brain tissue of MCAO/R mice, circ-FoxO3 activates autophagy in brain microvascular endothelial cells by inhibiting the expression level of mTORC1. Studies have found that circ-FoxO3 strengthens the connection between endothelial cells and reduces the damage of the blood–brain barrier by activating autophagy in brain microvascular endothelial cells [52].

#### 3.2.2. CircSHOC2

CircSHOC2 acts a miR-7670a-3p molecular sponge and was significantly upregulated in ischemic-preconditioned astrocyte-derived exosomes (IPAS-EXOs). MiR-7670a-3p was found to negatively regulate the expression of sirtuin 1 (SIRT1), resulting in a positive regulation of SIRT1 by circSHOC2 through the miR-7670a-3p/SIRT1 axis. SIRT1 can induce autophagy to promote neuronal cell survival [90]. The expression of SIRT1 was upregulated when circSHOC2 was enriched in neurons. However, circSHOC2 was not produced by nerve cells, but rather, delivered to nerve cells by astrocytes through exosomes. In conclusion, circSHOC2 in exosomes is transferred from astrocytes to neurons to activate the miR-7670a-3p/SIRT1 axis to reduce autophagy and inhibit apoptosis after ischemia/reperfusion injury [53].

### 3.3. Inflammation

Inflammation is a basic pathological process that occurs when tissues are stimulated by certain stimuli such as trauma, infection, and other damage factors. The blockage or rupture of blood vessels in the brain activates microglia to enter the infarct and penumbra regions. The expression of pro-inflammatory factors such as tumor necrosis factor (TNF), interleukin-1 (IL-1) and interleukin-6 (IL-6) is increased in the infarct region, and both infiltrating and resident cells jointly regulate the inflammatory response after stroke. Inflammation is a double-edged sword: it can promote cell death and also can relieve cerebral ischemia injury in stroke [91]. Previous studies have shown that circRNAs are involved in the regulation of inflammation after stroke.

#### 3.3.1. Circ_0000831

The expression of circ_0000831 was inhibited in the brain tissue of MCAO mice. As a miR-16–5p sponge, circ_0000831 promoted the expression of the downstream target adiponectin receptor 2 (AdipoR2). AdipoR2 can lead to the activation of peroxisome proliferator-activated receptor γ (PPARγ), which has been demonstrated in rats with cerebral ischemia to effectively inhibit neuroinflammation by suppressing the release of inflammatory factors [92]. Overexpression of circ_0000831 in both MCAO mice and the OGD model of BV-2 cells (microglia cells) resulted in a significant up-regulation of AdipoR2 and PPARγ expression, ultimately leading to the alleviation of neuroinflammation. Therefore, circ_0000831 can reduce neuroinflammation in cerebral ischemia through the miR-16–5p/ AdipoR2/PPARγ axis [54].

#### 3.3.2. CircDLGAP4

CircDLGAP4, located on exons 8, 9, and 10 of the DLGAP4 gene, was the sponge of miR-503–3p, which can downregulate the expression of neuronal growth regulator 1 (NEGR1). CircDLGAP4 can upregulate the expression of NEGR1 via miR-503–3p. In OGD-treated HCN-2 cells, the expression of circDLGAP4 was inhibited. After transferring circDLGAP4 into OGD-treated HCN-2 cells, NEGR1 was upregulated and the expression of miR-503–3p, TNF-α, IL-1β, and IL-6 was suppressed. Therefore, circDLGAP4 can inhibit inflammation and improve cell viability in OGD-treated HCN-2 cells through the miR-503–3p/NEGR1 axis [55]. Other studies have shown that circDLGAP4, acting as sponge of miR-142, alleviates blood–brain barrier damage after ischemic stroke by inhibiting endothelial transformation [93].

#### 3.3.3. CircRps5

CircRps5, which is abundant in the exosomes secreted by adipose-derived stem cells (ADSCs), was the sponge of miR-124–3p. MiR-124–3 negatively regulated Sirtuin 7 (SIRT7), a nicotinamide adenine dinucleotide oxidized-dependent deacetylase. By inhibiting miR-124–3p, circRps5 promoted SIRT7 expression. It has been shown that SIRT7 promoted the conversion of microglia from M1 to M2 phenotype, and the M2 phenotype of microglia releases anti-inflammatory factors to suppress inflammation [94]. In summary, circRps5 promotes the conversion of microglia from the M1 phenotype to the M2 phenotype through the miR-124–3/SIRT7 axis, releasing anti-inflammatory factors and reducing inflammation after stroke [56].

### 3.4. Oxidative Stress

Oxidative stress refers to an imbalance between the oxidation and antioxidation processes in the body often manifested in the accumulation of reactive oxygen species. It has negative effects, including the inflammatory infiltration of neutrophils, increased secretion of proteases, and the production of many oxidative intermediates generated by free radicals. Therefore, oxidative stress is considered to be an important factor in aging and disease. A large amount of oxygen free radicals was produced during cerebral ischemic injury, especially in the process of ischemia/reperfusion, and excessive oxygen free radicals could cause nerve cell dysfunction and even death. Inhibition of oxidative stress, which is a key process leading to brain injury in ischemic stroke, has been shown to have neuroprotective effects.

#### 3.4.1. Circ-Memo1

Circ-Memo1, which has been shown to be a potential therapeutic target for cancer, was upregulated in the peripheral blood of stroke patients in a recent study [57]. It was discovered that circ-Memo1 acts as a miR-17–5p sponge, regulating the expression of Son of Sevenless 1 (SOS1), which is inhibited by miR-17–5p. SOS1 could promote the phosphorylation of extracellular signal-regulating kinases 1/2 (ERK1/2) and activate the ERK/nuclear factor-κB (NF-κB) signaling pathway, which aggravates oxidative stress after stroke. Knocking out circ-Memo1 in hypoxia/reoxygenation(H/R)-treated HBMVECs cells increased the expression level of miR-17–5p and decreased the activity of SOS1, reducing oxidative stress and cell damage after stroke and improving cell viability. Evidently, knockdown of circ-Memo1 suppresses oxidative stress in H/R-treated HBMVECs through miR-17–5p/SOS1 axis [57].

#### 3.4.2. CircDLGAP4

AU-rich element RNA-binding protein 1 (AUF1) is a mammalian RNA-binding protein encoded by HNRNPD that regulates the translation and stability of the variety of mRNAs [95]. The nuclear factor erythroid 2-related factor 2 (NRF2)/heme oxygenase-1 (HO-1) signaling pathway inhibits cellular oxidative stress by decreasing the expression of intracellular reactive oxygen species (ROS). AUF1 forms a complex with circDLGAP4 that can subsequently interact with NRF2 mRNA, thereby promoting the enhancement of NRF2 mRNA stability. When circDLGAP4 was overexpressed in OGD-treated N2a cells, it promoted the expression of antioxidants such as NRF2 and HO-1 to inhibit oxidative stress [58].

#### 3.4.3. CircCTNNB1

CircCTNNB1 has been previously confirmed to be involved in tumor growth, invasion, and metastasis [96]. In a recent study, circCTNNB1 acted as the molecular sponge for miR-96-5p and positively regulated the downstream target scavenger receptor class B type 1 (SRB1). In MCAO/R mice, the expression of circCTNNB1 and SRB1 was suppressed, while miR-96-5p was upregulated. Interestingly, the upregulation of circCTNNB1 in MCAO/R mice led to a reduction in cerebral infarct size. When circCTNNB1 was upregulated in the OGD/R model of astrocytes, the expression of ROS and malondialdehyde (MDA) decreased, while superoxide dismutase (SOD) expression increased. Consequently, cellular oxidative stress was inhibited, leading to reduced cell damage. Moreover, pro-inflammatory factors in the cells were suppressed, mitigating the inflammatory response. Therefore, circCTNNB1 mitigates post-stroke oxidative stress and inflammatory response through the miR-96–5p/SRB1 axis [59].

### 3.5. Angiogenesis

Angiogenesis refers to the development of new blood vessels from existing capillaries or postcapillary veins, which is an important physiological process under the stimulation of hypoxia. After stroke, collateral circulation cannot provide the blood flow needed in the ischemic regions. Angiogenesis significantly improves blood flow and provides oxygen and nutrients to the ischemic regions [97].

#### 3.5.1. CircPDS5B

The CircPDS5B expression level was increased in the serum of tMCAO mice and stroke patients. Heterogenous nuclear ribonucleoprotein L (hnRNPL) belongs to the heteronuclear ribonucleoprotein family and is involved in RNA stability and transcriptional regulation [98]. Runt-related transcription factor-1 (Runx1) and Zinc finger protein 24 (ZNF24) are transcriptional inhibitors of vascular endothelial growth factor (VEGFA) [99]. It has been found that knocking down circPDS5B could reduce cerebral ischemic injury and promote angiogenesis. Mechanistically, circPDS5B recruits hnRNPL to enhance the stability of Runx1 and ZNF24 mRNA, promoting the expression of Runx1 and ZNF24 and inhibiting the transcription of VEGFA, thereby enhancing angiogenesis after stroke [60].

#### 3.5.2. CircHECTD1

CircHECTD1 was upregulated in the MCAO model of mice and in the OGD/R model of HBMECs. As a miR-335 sponge, it negatively regulated the expression of miR-335 [61]. Recent reports revealed that miR-335 can repress EndoMT and promote cell migration and tube formation via regulation of NOTCH2. Knockdown of circHECTD1 in both mouse MCAO model and HBMECs OGD model decreased NOTCH2 expression and promoted HBMECs migration and tube formation, while suppressing the EndoMT process [61].

#### 3.5.3. CircFUNDC1

In the OGD model of HBMECs, circFUNDC1 had a higher expression in the cytoplasm compared to the nucleus. Additionally, in patients with ischemic stroke, circFUNDC1 expression in peripheral blood was found to be upregulated [62]. CircFUNDC1 bound to miR-375 and inhibited its expression, leading to an increased expression level of the downstream target phosphatase and PTEN. Previous studies have shown that PTEN inhibited angiogenesis [100]. Silencing circFUNDC1 led to an increased expression level of miR-375 and an inhibition of PTEN. Consequently, angiogenesis in OGD-treated HBMECs was promoted. The up-regulation of Bcl-2 and VEGFA expression further facilitated the growth and migration of endothelial cells. Silencing circFUNDC1 can alleviate cell damage caused by oxygen and glucose deprivation and promote angiogenesis through the miR-375/PTEN axis [62].

#### 3.5.4. Circ_0006768

The miR-222–3p sponge, circ_0006768, is derived from zinc finger protein 292. It effectively inhibits the expression of miR-222–3p, thereby influencing cellular processes [63]. MiR-222–3p negatively regulates the expression of vascular endothelial zinc finger 1 (VEZF1). In previous studies, VEZF1 promoted angiogenesis by enhancing proliferation, migration, and vessel network formation [101]. As in OGD/R-treated HBNECs, circ_0006768 expression was suppressed in the plasma of ischemic stroke patients. Overexpression of circ_0006768 in OGD/R-treated HBNECs significantly induced the expression of VEZF1, leading to enhanced migration and tube formation abilities. In brief, circ_0006768 can promote the migration and tube formation abilities of HBNECs through the miR-222–3p/VEZF1 axis and reduce cell damage caused by OGD/R [63].

#### 3.5.5. CircPHKA2

The expression of circPHKA2 was inhibited in patients with acute cerebral ischemia and HBMECs treated with OGD. CircPHKA2 upregulated the expression of superoxide dismutase-2 (SOD2) via miR-574–5p by the molecular sponge effect. SOD2 is an antioxidant enzyme that plays an important role in maintaining vascular function [102]. In the OGD model of HBMEC, overexpression of circPHKA2 increased the expression of SOD2, leading to enhanced cell proliferation, migration, and tube formation, and reduced cell apoptosis. Ultimately, circPHKA2 promotes the angiogenesis ability of HBMECs and reduces cell damage through the miR-574–5p/SOD2 axis [64].

## 4. Potential Clinical Applications of circRNAs in Ischemic Stroke

CircRNAs play crucial roles in neurological disorders, such as ischemic stroke and neurodegenerative diseases. In ischemic stroke, circRNAs regulate gene expression, maintain BBB integrity, and serve as biomarkers. In other neurological disorders like Alzheimer’s and epilepsy, circRNAs contribute to diverse mechanisms, including protein aggregation, synaptic dysfunction, and altered neurotransmission [103]. The early pathological manifestation of Alzheimer’s disease involves the abnormal accumulation of Aβ in the cerebral cortex. CircHDAC9 was decreased in the serum of AD patients, acting as sponge of miR-138,which could increase miR-138 expression and reverse Sirt1 suppression and excessive Aβ production induced by miR-138 [104]. In Parkinson’s disease, circSV2b overexpression reduced oxidative stress damage, protected against the loss of dopaminergic neurons, maintained nigrostriatal function, and improved motor deficits through the miR-5107–5p-Foxk1-Akt1 signaling pathway [105]. CircSRRM4 binds with and hinders the participation of serine and arginine-rich splicing factor 3 (SRSF3) in the ubiquitin–proteasome pathway. This interaction enhances the SRSF3-mediated alternative splicing of pyruvate kinase M1/2, thereby promoting glycolysis to serve as an energy source in the context of epileptic seizures [106]. While circRNAs share common functions across disorders, their disease-specific roles, biomarker potential, and regulatory pathways vary. Understanding these distinctions is essential for developing targeted therapeutic approaches and precise diagnostic strategies in the field of neurology.

CircRNA expression is intricately regulated by the back-splicing machinery, involving RNA polymerases, transcription factors, and alternative splicing processes. At the same time, the back-splicing of circRNAs is regulated by cis-complementary sequences in flanking introns and by specific proteins [107]. In the context of the ischemic stroke, circRNAs exhibit dynamic expression changes, with certain circRNAs being upregulated or downregulated. These alterations are affected by the ischemia-induced microenvironment, characterized by oxygen and nutrient deprivation, inflammation, and oxidative stress. Compared to the sham surgery group, Li et al. found that, in mice seven days post-MCAO, there were 558 differentially expressed circRNAs in the ipsilateral thalamus. Furthermore, in mice 14 days post-MCAO, 2101 circRNAs exhibited significant changes in expression in the ipsilateral thalamus. Notably, 73 circRNAs showed significant alterations at both time points [108]. The degradation mechanism of circRNAs remains unclear at present, but there is evidence to suggest that endonucleases, Argonaute 2 (Ago2), N6-methyladenosine, and trimethylamine-n-oxide all play roles in this process. For instance, the binding of miRNA to circRNAs may lead to the degradation of circRNAs [109]. The turnover rate of CDR1as containing miR-671 binding sites depends on miR-671-directed cleavage by the slicer activity of Ago2 [109]. CircRNAs have been detected in various body fluids, including blood, saliva and cerebrospinal fluid, and their presence in extracellular fluids raises the possibility of using circRNAs as biomarkers for stroke diagnosis and prognostic. CircRNAs can be encapsulated in extracellular vesicles such as exosomes, and this packaging protects circRNAs from degradation by the extracellular environment, thereby maintaining their integrity and potential functionality as signaling molecules.

### 4.1. CircRNAs Act as Biomarkers for Ischemic Stroke

**Figure 2:**
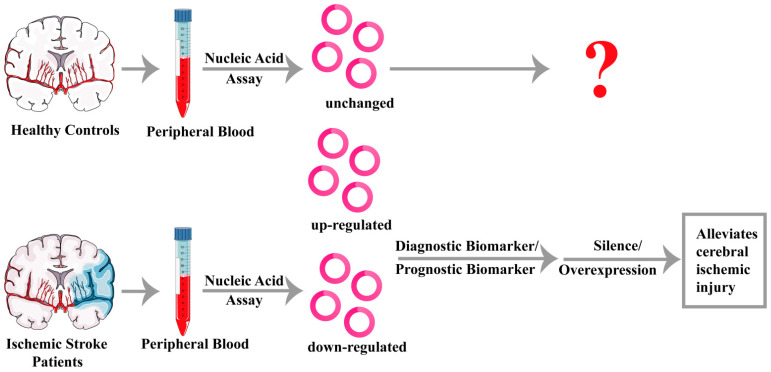
The potential clinical applications of circRNAs in stroke. Differential circRNA expression is examined by nucleic acid testing to diagnose and prognosis for ischemic stroke patients. CircRNAs are then silenced or over-expressed to alleviate cerebral ischemic injury. The function of circRNAs with unchanged expression is unclear in patients with ischemic stroke.

The expression of circRNAs differs between healthy individuals and ischemic stroke patients. Xu et al. studied the difference of circRNAs in exosomes between healthy and ischemic stroke patients in a Han Chinese population [110]. The study revealed that a total of 3540 circRNAs were abnormally expressed in exosomes of ischemic stroke patients compared with healthy individuals, among which 2363 circRNAs were downregulated and 1177 circRNAs were upregulated. Due to the circular structure of circRNAs and their transport in exosomes, they are not easily degraded by RNA exonucleases. Moreover, circRNAs can enter the body fluid circulation, which is more convenient to detect. The above reminds us to focus on the question of whether circRNAs could be used as highly specific markers for ischemic stroke (Figure 2).

CircRNAs have been shown to be potential biomarkers for the diagnosis of ischemic stroke caused by different triggers. For example, Li et al. identified that circ_0003574 was upregulated in patients with ischemic stroke compared with normal controls, and had a potential predictive and diagnostic value for ischemic stroke caused by intracranial atherosclerotic stenosis [111]. For diagnosing stroke caused by large artery atherosclerosis, Xiao et al. found that the expression levels of circ_0043837 and circ_0001801 had certain reference significance, and the circRNAs in exosomes had a better diagnostic value than those in plasma [112]. CircRNAs also have the potential to diagnose the degree of ischemia/reperfusion injury. In another study, the expression of circOGDH was significantly upregulated in the plasma of both MCAO mice and acute ischemic stroke patients, exhibiting an obvious correlation with its expression in brain tissue [32]. Partial regression analysis revealed a positive correlation between the area of penumbra and the level of circOGDH expression in the plasma of acute ischemic stroke patients. The studies above indicated that circRNAs hold potential as biomarkers for diagnosing stroke.

CircRNAs can be used not only as a diagnostic biomarker of stroke, but also as a prognostic biomarker. Zu et al. found that stroke patients with high circFUNDC1 expression had more severe neurological dysfunction and shorter survival than patients with low circFUNDC1 expression [113]. In addition, Zuo et al. found that within seven days after stroke, the expression trend of circFUNDC1, circPDS5B, and circCDC14A in patients with good prognosis is opposite to that in patients with severe prognosis [114]. The greater the changes in the expression of circRNAs, the worse the prognosis of the patients when compared to patients with small changes. CircRNAs exhibit tissue-specific expression patterns, including in the brain and central nervous system. This specificity suggests that circRNAs could serve as biomarkers for ischemic stroke or even different disease stages. Comparative analysis of circRNAs expression profiles between healthy individuals and patients could identify ischemic stroke-associated circRNAs and help differentiate between different subtypes or stages of ischemic stroke.

The detection and quantification of circRNAs in clinical settings involve several techniques. Reverse transcription quantitative polymerase chain reaction (RT-qPCR) is a widely used method that allows for the specific amplification and quantification of circRNAs. However, designing circRNA-specific primers can be challenging because their sequences often overlap with linear counterparts. High-throughput RNA sequencing (RNA-seq) provides a comprehensive approach to circRNA profiling, enabling the identification of novel circRNAs and the assessment of their expression levels. Additionally, microarray-based techniques provide a high-throughput platform for circRNA expression analysis. Recent advances in molecular technology have facilitated the development of more sensitive and specific methods for circRNA detection. For instance, Northern blotting and RNase R treatment, which selectively degrades linear RNA, can be employed to confirm the circular nature of candidate circRNAs. Furthermore, the use of molecular beacons, a type of nucleic acid probe, allows for the real-time imaging of circRNA expression in living cells. In situ hybridization (ISH) techniques, such as fluorescence in situ hybridization (FISH), provide spatial information about circRNAs localization within tissues. The development of detection and quantification technologies promotes the potential of circRNAs as biomarkers.

### 4.2. CircRNAs Act as Treatment Targets for Ischemic Stroke

CircRNAs can be targeted for the treatment of stroke (Figure 2). As mentioned above, circRNAs are involved in regulating various pathological activities after stroke. Silencing or promoting the expression of specific circRNAs can improve cerebral ischemia/reperfusion injury and neurological function recovery. For instance, Yang et al. discovered that by injecting lentivirus containing circ-FoxO3 into the lateral ventricles of MCAO/R mice, BBB damage can be attenuated [52]. Furthermore, if a drug can influence circRNAs and prevent their binding to miRNA or RNA-binding proteins, the sponge role of circRNAs can be inhibited. Chen et al. found that Buyang Huanwu Decoction can reduce cerebral ischemic injury through the circRNA-miRNA-mRNA network [115].

CircRNAs can enter body fluid circulation through the exosome pathway and reach target cells to exert biological functions. Exosomes are membrane vesicles with a diameter of 30–150 nm that are released into the extracellular space after the fusion of multivesicular bodies formed by the invagination of lysosomal microparticles and cytomembrane. Studies have found that exosomes secreted by neuronal cells, astrocytes, microglia, oligodendrocytes, and vascular endothelial cells attenuated cerebral ischemic injury [116]. Exosomes are widely distributed in body fluids such as plasma, saliva, cerebrospinal fluid, breast milk, tears, lymph, bile, and gastric acid, and can penetrate the BBB [117]. After entering the body fluid, exosomes can be directionally delivered to target cells [118], thus allowing efficient delivery of circRNAs to target cells. Chen et al. discovered that exosomes with circSHOC2 released from astrocytes after stroke could inhibit neuronal apoptosis and alleviate cerebral ischemic injury [53]. The specific delivery of circRNAs to target cells by exosomes reminds us that circRNAs can be encapsulated in exosome-like extracellular vesicles to deliver circRNAs to brain tissue in vitro, ultimately reducing cerebral ischemic injury. Yang et al. engineered brain-targeted extracellular vesicles containing circSCMH1 to promote functional recovery after cerebral ischemia in rodents and nonhuman primates by intravenous injection [119].

## 5. Conclusions and Perspective

**Figure 3:**
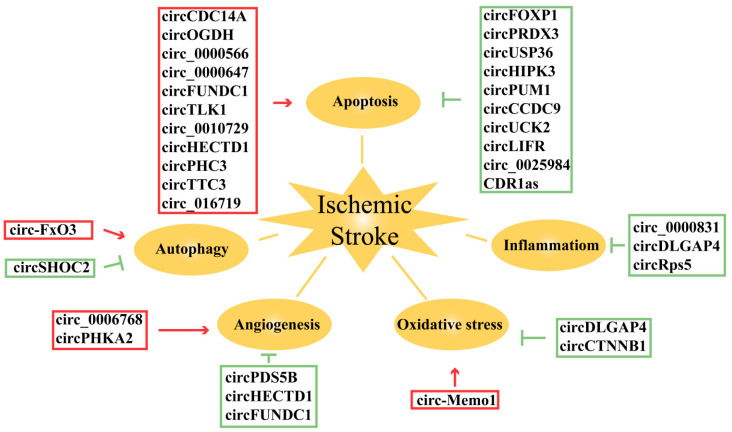
Summary of circRNAs involved in ischemic stroke pathophysiology.

In this review, we initially outlined the five biological functions of circRNAs to gain a better understanding of their role in stroke. Next, we summarized the roles of 31 circRNAs in five pathophysiological processes of ischemic stroke and explained the signaling pathways associated with each circRNA (Figure 3). Furthermore, we discussed the potential clinical applications of circRNAs as therapeutic targets and biomarkers.

As has been established, circRNAs, as sponges for miRNA, are involved in the process of apoptosis, autophagy, inflammation, oxidative stress, and angiogenesis after ischemic stroke. However, there is still a lack of understanding regarding the mechanisms by which circRNAs contribute to ischemic stroke, such as their ability to translate proteins, regulate transcription, bind to RNA-binding proteins, or act as protein scaffolds. Additionally, the changes in circRNA expression after ischemic stroke and the mechanism of circRNA degradation are not yet clear. At present, most research on circRNAs in stroke disease is limited to preclinical studies. Targeting circRNAs in ischemic stroke therapy offers significant promise, given their regulatory roles in key pathways associated with ischemic stroke pathophysiology. However, innovative delivery mechanisms are crucial due to the BBB restrictions: tools such as exosome delivery, nanoparticles, synthetic circRNAs, and CRISPR technologies offer promising avenues. CircRNA overexpression vectors based on intronic complementary sequences may induce mis-splicing, leading to nonspecific and potentially harmful effects. Current vectors cannot generate target circRNAs without mis-spliced products. While purified in vitro synthesized circRNA molecules could address these issues, challenges with large-scale synthesis may restrict the therapeutic potential of synthetic circRNAs. However, synthetic circRNAs can induce immune system activation in vivo. In conclusion, while targeting circRNAs in ischemic stroke holds therapeutic potential, ongoing research is essential to overcome challenges and fully harness their benefits in stroke treatment.

Ischemic stroke is often diagnosed after the onset of the disease, and there is a lack of specific biomarkers similar to those of other diseases that can signify the occurrence of ischemic stroke in advance. Currently, clinical magnetic resonance imaging (MRI) technology is used to detect penumbral areas and usually takes about a half hour. On the other hand, nucleic acid testing provides a much faster diagnosis with lower requirements for testing location. It can even be performed at home, which is fortunate for ischemic stroke patients with a narrow rescue time-window. Therefore, it is urgent for us to find a suitable circRNA as the “gold standard” for the nucleic acid testing of ischemic stroke.

Among the currently known biological functions of circRNAs, the translation into proteins, regulation of transcription, binding with RNA-binding proteins, and their ability to act as protein scaffolds remain poorly understood. Additionally, the changes in circRNA expression and the mechanisms of circRNA degradation following ischemic stroke are still unclear. Currently, most research on circRNAs in stroke diseases is limited to preclinical studies. Future investigations may focus on validating the safety and effectiveness of extracellular vesicle-mediated circRNA delivery in vivo. Exploring strategies to reduce the immunogenicity of synthesized circRNAs, including introducing chemical modifications and encapsulating them within RNA-binding proteins, could be an avenue of interest.

CircRNAs have demonstrated promising prospects in the biomarkers and treatment of ischemic stroke. Continuous research on circRNAs in ischemic stroke is highly recommended.

## Figures and Tables

**Table 1:** The expression and targets of stroke associated circRNAs.

Phenotype	CircRNA	Animals and/or Cells	Model	Expression	Targets	The Roles in Stroke	References
Apoptosis	CircFOXP1	Mice, A172 cells	tMCAO, OGD/R	Decreased	STAT3	Alleviation	Yang et al. [30]
	CircCDC14A	Mice, HT22 cells	MCAO/R, OGD/R	Increased	miR-23a-3p	Aggravation	Huo et al. [31]
	CircOGDH	Mice	MCAO	Increased	miR-5112	Aggravation	Liu et al. [32]
	CircPRDX3	Mice, N2a cells	MCAO/R, OGD/R	Decreased	miR-641	Alleviation	Chen et al. [33]
	Circ_0000566	HBMECs	OGD/R	Increased	miR-18a-5p	Aggravation	Liu et al. [34]
	Circ_0000647	SK-N-SH cells	OGD/R	Increased	miR-126-5p	Aggravation	Dai et al. [35]
	CircFUNDC1	HCN-2 cell, HBMECs	OGD	Increased	miR-496	Aggravation	Wang et al. [36]
	CircTLK1	Mice, N2a cells	MCAO/R, OGD/R	Increased	miR-26a-5p	Aggravation	Wu et al. [37]
	CircUSP36	Mice, A172 cells	MCAO/R, OGD/R	Decreased	miR-139-3p	Alleviation	Yang et al. [38]
	CircHIPK3	Mice, bEnd.3 cells	tMCAO, OGD/R	Decreased	miR-148b-3p	Aggravation	Chen et al. [39]
	Circ_0010729	HBMECs	OGD/R	Increased	miR-665	Aggravation	Ouyang et al. [40]
	CircPUM1	Mice, SK-N-SH cells	tMCAO, OGD/R	Decreased	miR-340-5p	Alleviation	Hu et al. [41]
	CircHECTD1	Mice, HT22 cells	MCAO, OGD	Increased	miR-133b, miR-27a-3p	Aggravation	Zhang et al. [42], Dai et al. [43]
	CircPHC3	HBMECs	OGD	Increased	miR-455-5p	Aggravation	Xu et al. [44]
	CircTTC3	Mice, Primary astrocytes	MCAO/R, OGD/R	Increased	miR-372-3p	Aggravation	Yang et al. [45]
	CircCCDC9	Mice	tMCAO	Decreased	Notch1, NICD	Alleviation	Wu et al. [46]
	CircUCK2	Mice, HT22 cells	MCAO, OGD	Decreased	miR-125b-5p	Alleviation	Chen et al. [47]
	Circ_016719	Mice, HT22 cells	tMCAO, OGD/R	Increased	miR-29c	Aggravation	Tang et al. [48]
	CircLIFR	Mice, bEnd.3 cells	MCAO, OGD	Decreased	miR-100	Alleviation	Zhao et al. [49]
	Circ_0025984	Rats, A172 cells	MCAO, OGD	Decreased	miR-134-3p	Alleviation	Zhou et al. [50]
	CDR1as	Mice	MCAO/R	Decreased	miR-7	Alleviation	Mehta et al. [51]
Autophagy	Circ-FoxO3	Mice, bEnd.3 cells and HBMECs	MCAO/R, OGD/R	Increased	mTOR, E2F1	Alleviation	Yang et al. [52]
	CircSHOC2	Primary astrocytes	OGD/R	Increased	miR-7670a-3p	Alleviation	Chen et al. [53]
Inflammation	Circ_0000831	Mice, BV-2 cells	MCAO, OGD	Decreased	miR-16-5p	Alleviation	Huang et al. [54]
	CircDLGAP4	HCN-2 cells	OGD	Decreased	miR-503-3p	Alleviation	Qiu et al. [55]
	CircRps5	Mice, ADSCs	MCAO, OGD	Increased	miR-124-3	Alleviation	Yang et al. [56]
Oxidative stress	Circ-Memo1	HBMECs	H/R	Increased	miR-17-5p	Aggravation	Ren et al. [57]
	CircDLGAP4	Mice, N2a cells	tMCAO, OGD	Decreased	AUF1	Alleviation	Liu et al. [58]
	CircCTNNB1	Mice, Primary astrocytes	MCAO/R, OGD/R	Decreased	miR-96-5p	Alleviation	Chen et al. [59]
Angiogenesis	CircPDS5B	Mice, HBMECs	tMCAO, OGD/R	Increased	hnRNPL	Aggravation	Jiang et al. [60]
	CircHECTD1	Mice, HBMECs	MCAO, OGD/R	Increased	miR-335	Aggravation	He et al. [61]
	CircFUNDC1	HBMECs	OGD	Increased	miR-375	Aggravation	Bai et al. [62]
	Circ_0006768	HBNECs	OGD/R	Decreased	miR-222-3p	Alleviation	Li et al. [63]
	CircPHKA2	HBMECs	OGD	Decreased	miR-574-5p	Alleviation	Yang et al. [64]

CircRNA: circular RNA; A172 cells: Human Glioblastoma cells; HT22 cells: Hippocampal Neuronal cell line; N2a cells: Neuro2a cells; HBMECs: Human Brain Microvascular Endothelial cells; SK-N-SH cells: Human Neuroblastoma cell line; bEnd.3 cells: brain-derived Endothelial cells.3; BV-2 cells: microglia cells line; HCN-2 cells: Human Cortical Neurons; ADSCs: Adipose-Derived Stem cells; STAT3: Signal Transducer and Activator of Transcription 3; NICD: Notch intraCellular domain; mTOR: mammalian target of rapamycin; E2F1: E2F transcription factor 1; AUF1: AU-rich element RNA-binding protein 1; hnRNPL: heterogenous nuclear ribonucleoprotein L.
